# Does the degree of intraoperatively identified cartilage loss affect the outcomes of primary total knee arthroplasty without patella resurfacing? A prospective comparative cohort study

**DOI:** 10.1186/s43019-022-00161-3

**Published:** 2022-07-18

**Authors:** Oog-Jin Shon, Gi Beom Kim

**Affiliations:** Department of Orthopedic Surgery, Yeungnam University College of Medicine, Yeungnam University Medical Center, Hyeonchungno 170, Nam-gu, Daegu, 42415 Republic of Korea

**Keywords:** Knee, Total knee arthroplasty, Patellar resurfacing, Patellar retention

## Abstract

**Purpose:**

The aim of this study was to investigate whether the degree of patellar cartilage loss confirmed during index surgery affects the clinical and radiologic outcomes of total knee arthroplasty (TKA) performed without patellar resurfacing.

**Methods:**

We prospectively divided 2012 patients with a minimum follow-up of 12 months into two groups according to intraoperatively graded cartilage lesions graded using the International Cartilage Repair Society (ICRS) system: group 1, grades 0‒2 (*n* = 110); group 2, grades 3‒4 (*n* = 102). Relevant locations, such as medial, lateral, or both facets of the patella, were also assessed. Clinical outcomes were assessed using the Western Ontario and McMaster Universities Osteoarthritis Index, Feller’s patella score, and Kujala anterior knee pain score. Radiographic outcomes included patellar tilt angle and lateral patellar shift on Merchant’s view.

**Results:**

Clinical and radiographic outcomes were not significantly different between the two groups. No patient underwent secondary patellar resurfacing. Although the lateral facet was significantly more involved, there were no significant differences in outcomes.

**Conclusions:**

The degree of intraoperatively identified patellar cartilage loss did not affect the short-term outcomes following primary TKA without patellar resurfacing.

*Level of evidence* II: Prospective comparative study.

## Introduction

Patellar resurfacing has been performed in approximately 35% of international joint registries during primary total knee arthroplasty (TKA) [1]. However, the management of the patella remains controversial. Although some surgeons have advocated for patellar resurfacing in terms of decreased postoperative anterior knee pain and risk of progressive patellar cartilage loss [2], many studies have reported that this procedure does not necessarily show superior outcomes [3–5]. Moreover, there is a lack of evidence that patellar cartilage loss identified during TKA causes anterior knee pain or decreased function [6]. Some studies have reported that there were no differences in outcomes between patients who underwent or did not undergo patellar resurfacing, regardless of the degree of patellar cartilage loss [7–9]. The authors of one study reported satisfactory radiologic outcomes with patellar retention in patients with grade 4 advanced osteoarthritis (OA) in the patellofemoral joint [10].

In addition, the reporting of adverse events associated with patellar resurfacing, including periprosthetic fracture, aseptic loosening, and infection, have recently led many surgeons to advocate patellar retention [6, 7, 10].

Although some retrospective studies have reported favorable outcomes of patellar retention during primary TKA [6, 7], there is a paucity of literature on the prospective outcomes of this procedure. Therefore, the purpose of this prospective study was to investigate whether the degree of patellar cartilage loss confirmed during index surgery affects the short-term clinical and radiologic outcomes of TKA performed without patellar resurfacing. Moreover, we sought to assess the differences in outcomes according to the involved facet of the patella. To this end, we asked two questions: (1) Does the degree of patellar cartilage loss identified during surgery affect the outcomes after primary TKA without patellar resurfacing? (2) Do the outcomes differ depending on the location of the involved facet of the patella? We hypothesized that patellar cartilage loss has no effect on the short-term follow-up outcomes in primary TKA. We also hypothesized that there would be no difference in outcomes depending on the location of the facet involved.

## Methods

### Patient demographic characteristics

**Fig. 1:**
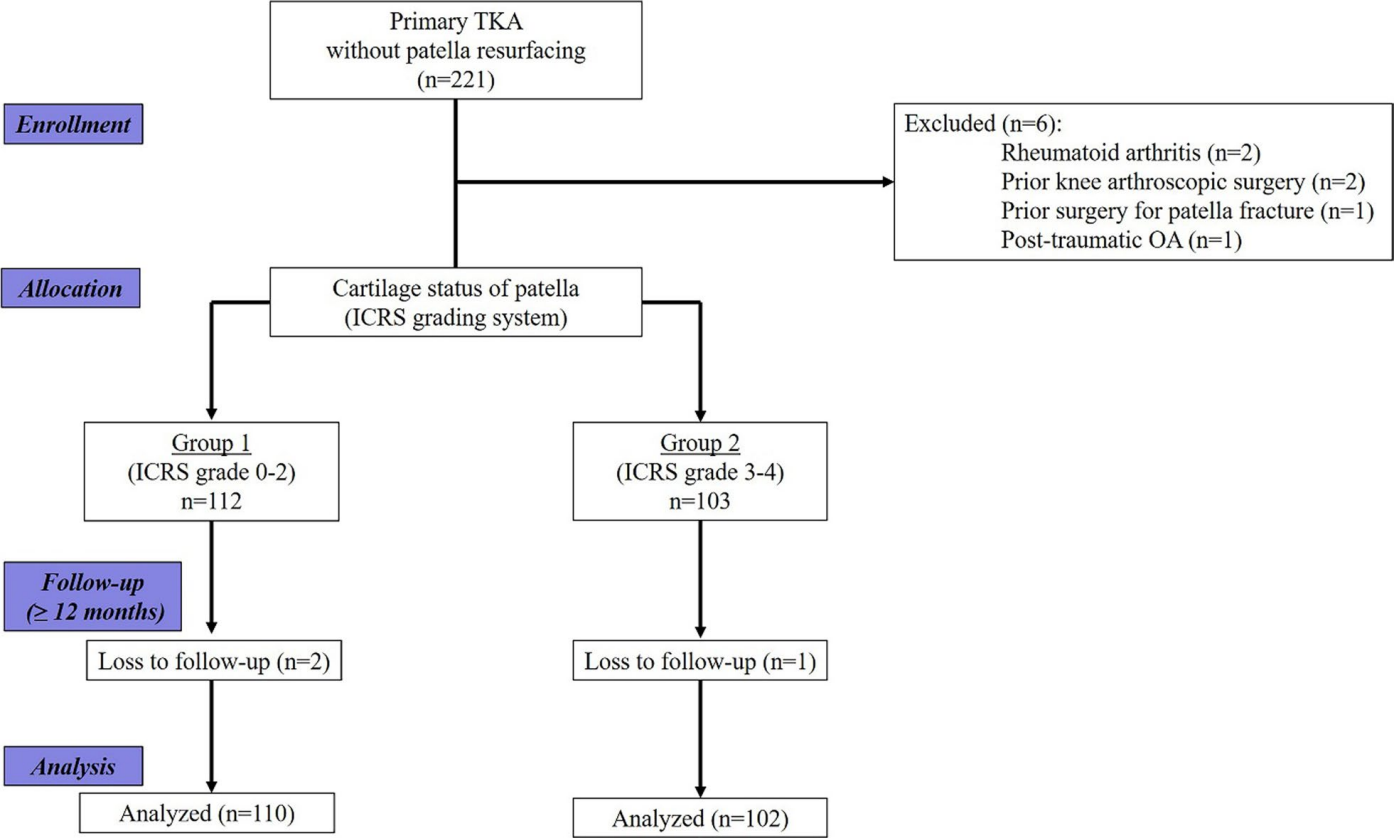
Flow diagram of patient enrollment.* n* = number of knees.* ICRS* International Cartilage Repair Society,* OA* osteoarthritis,* TKA* total knee arthroplasty

All patients participating in this study were asked for informed consent prior to enrollment. This prospective cohort study was approved by the Institutional Review Board of our hospital before patient data were retrieved (YUMC 2019-06-062-001). From July 2019 to April 2020, 221 knees which underwent consecutive primary TKA without patellar resurfacing were screened. We enrolled patients who were prospectively eligible for clinical and radiographic assessments with a minimum follow-up of 12 months after the index surgery. Patients with inflammatory arthritis (e.g., rheumatoid arthritis), previous surgery around the knee (e.g., arthroscopy, open reduction and internal fixation due to patellar fracture), and post-traumatic OA were excluded. Of the nine knees excluded, three were lost to follow-up, two had rheumatoid arthritis, two underwent arthroscopic surgery, one underwent surgery for patellar fracture, and one had post-traumatic OA. Among those patients who met the inclusion criteria, patients were classified according to the intraoperatively confirmed patella cartilage loss based on the International Cartilage Repair Society (ICRS) grading system [11]. Finally, to meet the minimum number of patients required for each group through the sample size calculation, we enrolled 212 knees in the study (Fig. 1).

### Surgical techniques

All surgeries were performed by a senior surgeon using the same technique, namely, the modified gap-balancing technique which can balance the extension gap before the flexion gap using the posterior-stabilized gradually reducing radius femoral implant (Attune™; Depuy Synthes, Warsaw, IN, USA) [12, 13]. A medial parapatellar arthrotomy with a midline incision was performed. Femur sizing was performed using an anterior reference system in all cases. The rotation of the tibial component was set by considering several reference points, including the medial one-third of the tibial tuberosity, anterior tibial cortex, and floating technique. All prostheses were used with cement. Antioxidant polyethylene inserts were used in all cases.

No resurfacing of the patella occurred during surgery, regardless of the degree of patellar arthritic change and the original thickness of the patella. Patelloplasty was performed, which included the removal of marginal osteophytes, flattening of denuded facets, and circumferential denervation using electrocautery. Intraoperative patellar tracking was checked throughout the knee motion with the no thumb technique [14, 15].

A single closed suction drain was inserted after surgery and removed 24 h later. The perioperative pain control protocol was identical for all patients, including multimodal drug regimen, postoperative patient-controlled analgesia, and intraoperative periarticular injection. Active dangling exercise was initiated on the day of surgery, and partial weight-bearing was allowed on the first postoperative day. Full weight-bearing was permitted 3 weeks after surgery.

### Intraoperatively confirmed cartilage loss of patella and grouping

**Fig. 2:**
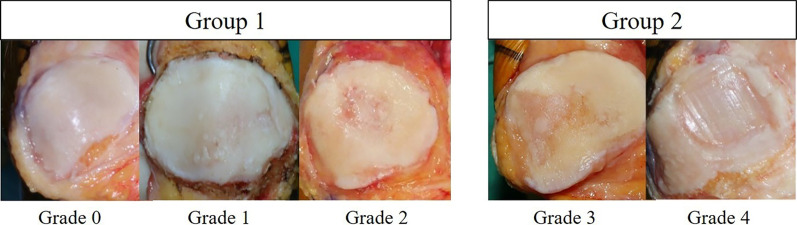
Patients’ assignment according to ICRS grading system. Group 1 patients had normal to mild cartilage loss, with ICRS grades 0‒2. Group 2 patients had moderate to severe cartilage loss (ICRS grades 3‒4)

The degree of cartilage loss of the patella was independently assessed by two orthopedic knee specialists during surgery using the ICRS grading system [11]. An assistant who was not involved in the operation recorded the assessment of each surgeon. If there was a disagreement between the two experts, the third orthopedic knee specialist made the final decision on the grading. We performed baseline surveys for the prospective collection of data with two concurrent cohorts of patients who were assigned to either group 1 (normal to mild cartilage loss; ICRS grades 0‒2; *n* = 110) or group 2 (moderate to severe cartilage loss, ICRS grades 3‒4, *n* = 102) (Fig. 2). For subgroup analysis, relevant locations, such as medial, lateral, or both facets of the patella, were assessed.

### Outcome measures

Clinical and radiographic outcomes of each patient were assessed before surgery, at 6 weeks after surgery, and at 3, 6, and 12 months after surgery. Clinical outcomes were assessed using the Western Ontario and McMaster Universities Osteoarthritis Index (WOMAC) score [16], Feller’s patella score [17], and the Kujala Anterior Knee Pain (AKP) score [18]. Reoperation related to secondary patellar resurfacing was also evaluated.

Radiographic outcomes included the patellar tilt angle and lateral patellar shift on Merchant’s view radiograph [19–21]. Lateral patellar tilt was defined as the angle between the line crossing the widest portion of the patella and the line passing through the anterior surfaces of both condyles. Lateral patellar shift was defined as the distance between the anterior-most point of the lateral femoral condyle and a line from the lateral edge of the patella perpendicular to the line that passed through the anterior surface of both condyles.

### Statistical analysis

A power analysis (G* power software, version 3.1.7) was performed to calculate the number of patients needed in each group to identify the significant differences in clinical outcomes [power of 90%, α error of 0.05, and standard deviation (SD) of 10]. Eighty-five patients per group were required to identify significant differences. Ultimately, 102 patients were assigned to each group taking into account an estimated loss to follow-up of approximately 20%.

Statistical evaluation was performed using IBM SPSS software version 23 (IBM Corp., Armonk, NY, USA), and continuous data were expressed as the mean ± SD. All dependent variables were tested for normality of distribution and equality of variances using the Kolmogorov–Smirnov test and analyzed using parametric or non-parametric tests based on normality. An independent samples *t*-test (parametric) and Mann–Whitney *U*-test (non-parametric) were performed to assess the differences in clinical and radiographic variables between the two groups. Fisher’s exact test was used to compare the ratios between the groups. For subgroup analysis, the Kruskal–Wallis test was used to assess the differences among the three groups. Statistical significance was set at *p* < 0.05.

Reliabilities for the degree of cartilage loss were analyzed using intraclass correlation coefficients, and reliabilities were classified as little, if any (correlation coefficient ≤ 0.25), low (0.26–0.49), moderate (0.50–0.69), high (0.70–0.89), or very high (≥ 0.90) [22].

## Results

The average age at surgery was 71.6 (range 60.0‒85.0) years, and the average follow-up period was 16.2 (range 12.0‒21.0) months. There were no significant differences in the demographic variables between the groups (Table 1).

**Table 1:** Patient demographic characteristics

Patient demographic characteristics	Overall (*n* = 212)	Group 1 (*n* = 110)	Group 2 (102)	*p* value^a^
Age (years)	71.6 (60.0–85.0)	71.8 (60.0–85.0)	71.3 (61.0–82.0)	0.692
*Sex, n (%)*				
Female	191 (90.1)	102 (93.6)	89 (86.4)	0.091
Male	21 (9.9)	7 (6.4)	14 (13.6)	
BMI (kg/m^2^)	26.7 (19.5–32.3)	26.9 (19.5–30.5)	26.3 (20.1–32.3)	0.725
F/U period (months)	16.2 (12.0–21.0)	16.3 (12.0–21.0)	16.2 (12.0–20.0)	0.641
*Side, n (%)*				
Right	97 (45.8)	51 (46.8)	46 (44.7)	0.681
Left	115 (54.2)	58 (53.2)	57 (55.3)	0.626
Preop HKA angle (°)^b^	− 5.5 (− 25 to 16.5)	− 5.9 (− 25 to 13.5)	− 5.2 (− 20 to 16.5)	0.735
*Preop ROM*				
FC (°)^c^	9.5 (− 10.0 to 35.0)	9.5 (− 10.0 to 25.0)	9.5 (− 8.0 to 35.0)	0.607
MF (°)	115.5 (70.0–150.0)	118.5 (70.0–150.0)	112.5 (80.0–150.0)	0.719

*BMI* Body mass index, *F/U* follow-up, *Preop* preoperative, *HKA angle* hip-knee-ankle angle, *ROM* range of motion, *FC* flexion contracture, *MF* maximal flexion

Data are presented as the mean with the range in parentheses, unless indicated otherwise

^a^The level of statistical significance was set at *p* < 0.05

^b^Varus alignment is indicated as HKA angle < 0°

^c^Negative value of FC indicates hyperextension

**Fig. 3:**
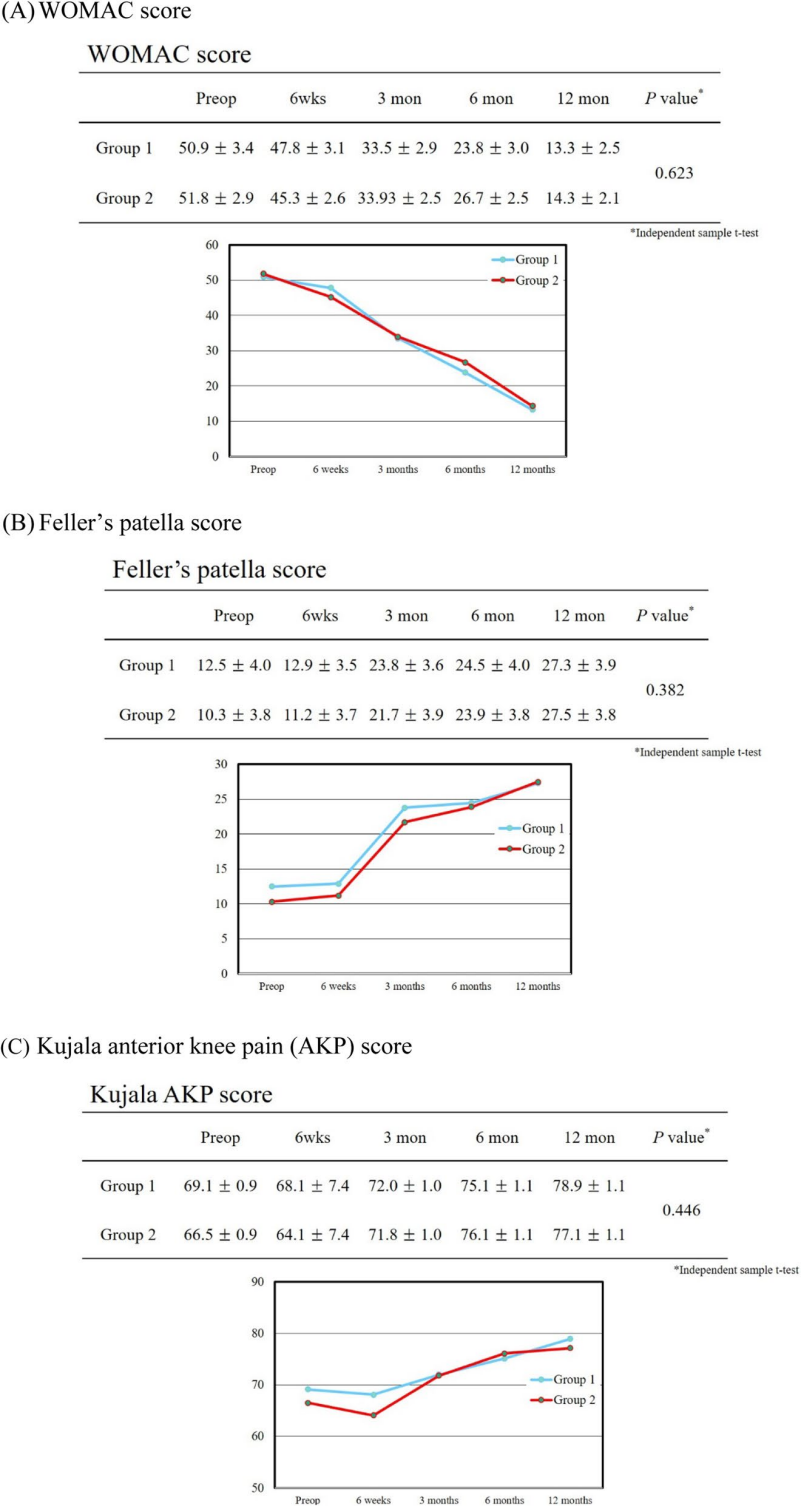
Clinical outcomes before surgery, at 6 weeks after surgery, and at 3, 6, and 12 months following TKA without patellar resurfacing. No statistically significant differences were observed between the two groups according to the Western Ontario and McMaster Universities Osteoarthritis Index (*WOMAC*) score, Feller’s patella score, and the Kujala Anterior Knee Pain (*AKP*) score.* Preop* Preoperative

**Fig. 4:**
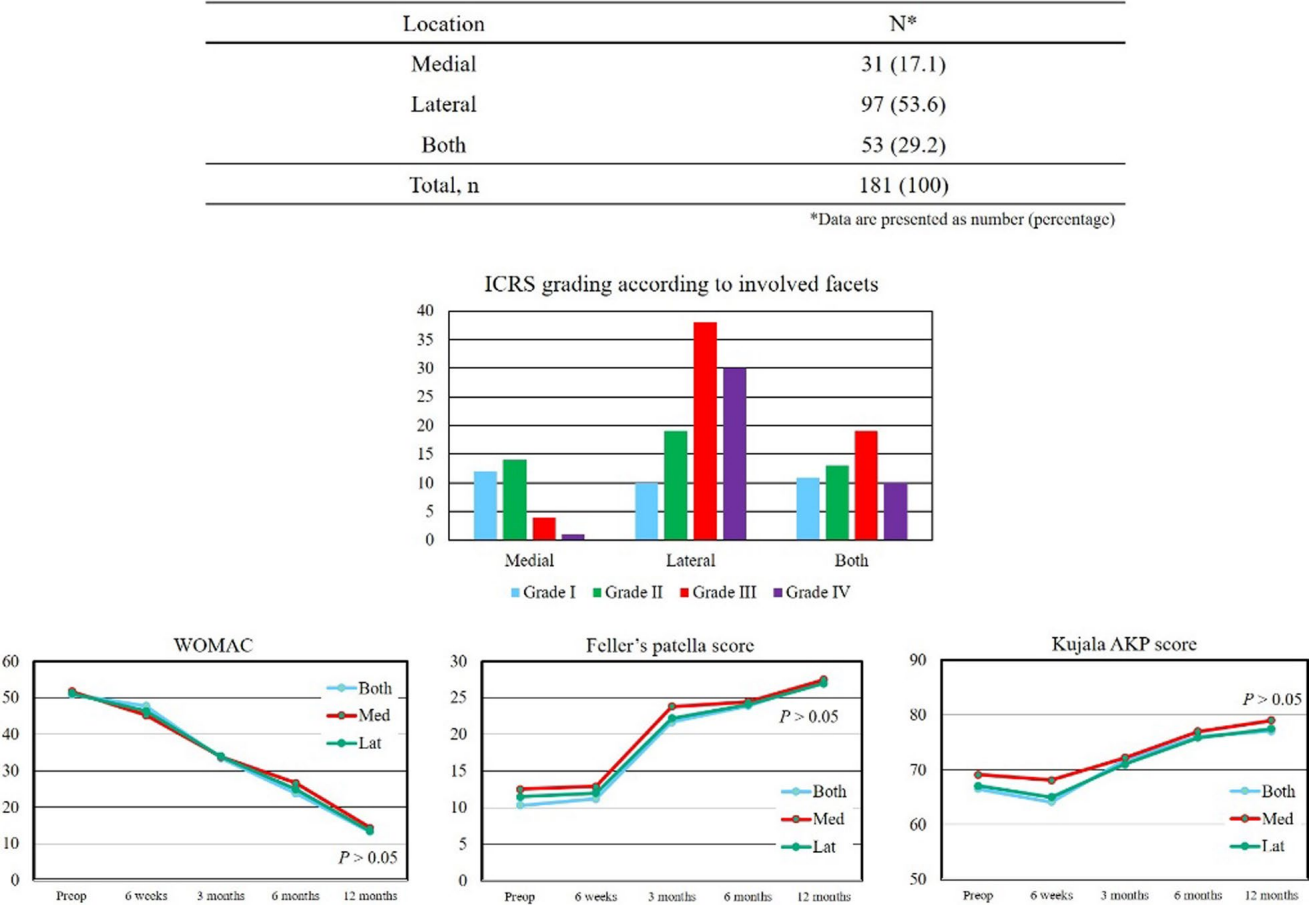
Subgroup analysis showed relevant locations of the patella (medial, lateral, and both facets). No significant differences were observed in clinical outcomes among the involved facets

Patellar cartilage loss was the highest in patellas assessed with ICRS grade 3 (28.8%) (Table 2). At 12 months after surgery, clinical outcomes, including WOMAC, Feller’s patella score, and Kujala AKP score, were not significantly different between the two groups (Fig. 3). After the index surgery, no significant difference was observed in radiographic outcomes, and there was no significant change during follow-up (Table 3). During the study period, no patient complained of anterior knee pain (AKP), and none of the patients underwent secondary patellar resurfacing. Although subgroup analyses were significantly more lateral among the relevant facets, they did not show significant differences in clinical outcomes (Fig. 4). Intraoperative agreement of the cartilage status showed very high intra- and inter-observer reliabilities (Table 4).

**Table 2:** Intraoperative measurements of patella showing cartilage loss according to the ICRS grading system

ICRS grade	Overall cohort (*n* = 212)	Group 1 (*n* = 110)^a^	Group 2 (*n* = 102)^a^
0	31 (14.6)	110 (51.9)	–
1	33 (15.6)		–
2	46 (21.7)		–
3	61 (28.8)	–	102 (48.1)
4	41 (19.3)	–	
Total, *n*	212 (100)		

Data are presented as a number with the percentage in parentheses

^a^Group 1, ICRS grade 0–2. Group 2, ICRS grade 3–4

**Table 3:** Radiographic outcomes

Radiographic outcomes	Before surgery	*p* value^a^	6 weeks	*p* value^a^	3 months	*p* value^a^	6 months	*p* value^a^	12 months	*p* value^a^
*Patella tilt angle*										
Group 1	6.1 ± 4.2	0.719	5.8 ± 3.9	0.794	5.8 ± 3.8	0.813	5.9 ± 3.9	0.852	5.9 ± 4.0	0.835
Group 2	7.9 ± 4.7		6.0 ± 4.0		6.0 ± 3.9		6.0 ± 3.9		6.0 ± 4.0	
*Lateral patella shift*										
Group 1	6.8 ± 2.2	0.128	5.3 ± 2.5	0.219	5.2 ± 2.6	0.269	5.3 ± 2.5	0.254	5.3 ± 2.4	0.293
Group 2	8.7 ± 3.1		6.2 ± 3.3		6.3 ± 3.4		6.2 ± 3.3		6.2 ± 3.1	

Data are presented as the mean ± standard deviation

^a^Independent sample *t*-test. The level of statistical significance was set at *p* < 0.05

**Table 4:** Intra- and inter-class correlation coefficients of the intraoperative cartilage status of the patella

Grade	Intra-observer	Inter-observer
Grade 0	0.98	0.96
Grade 1	0.96	0.93
Grade 2	0.93	0.91
Grade 3	0.92	0.91
Grade 4	0.98	0.95

Values are presented as absolute values. The data show almost perfect intra- and inter-observer agreement in the measured parameters [23]

## Discussion

The most notable finding of this study was that patients who underwent primary TKA without patellar resurfacing did not show significant differences in clinical and radiographic outcomes up to 2 years after surgery, regardless of the degree of patellar cartilage loss.

To the best of our knowledge, there is as yet no consensus on the optimal management of the patella during primary TKA. Some authors have advocated for a resurfacing procedure for patellas with an almost denuded cartilage [23, 24], while others have reported that patellar resurfacing fails to guarantee better outcomes even with deterioration of patellar cartilage [7, 8, 10]. In this study, we did not perform resurfacing even in those patellas with almost denuded cartilage. This decision was primarily due to the surgeon’s choice based on our hypothesis in this study.

Several adverse events associated with patellar resurfacing have been reported, including patellar fracture, osteonecrosis, extensor mechanism malalignment, and loosening or wear of the patellar component [25–27]. Moreover, some studies have reported that it was very difficult to restore patellar thickness and adequate patellar tracking [28]. Inappropriate patellar thickness may also affect patellofemoral overstuffing [29, 30], and a resultant thickness of < 12 mm after resection has an inherent risk of periprosthetic fracture [31–33].

Furthermore, with the exception of clinical and radiographic outcomes, our results showed that no patients complained of AKP or underwent additional secondary patella surfacing during an average follow-up period of 16.2 months. Some studies have reported an increased incidence of AKP after primary TKA in the absence of resurfacing [34, 35] and suggested secondary resurfacing as a rescue procedure [36, 37]. However, since AKP has multifactorial characteristics, it has been reported that the effect of secondary resurfacing is only 40‒50% relief of the symptoms [36]. The possibility of developing patella cartilage loss after primary TKA may also be a concern [38]. However, to the best of our knowledge, there is a paucity of literature on the subject of patella cartilage loss that develops several years after TKA; even if patella cartilage loss is progressive, it is difficult to conclude that such deterioration is associated with inferior clinical outcomes or worsening of AKP.

Despite the informative results of this study, the study does have a number of limitations that need to be considered. First, the relatively short follow-up period may be a major concern. Significant differences may have been missed because mid- to long-term outcomes were not assessed. However, some studies have reported that gradual cartilage loss of the patella was not necessarily observed after primary TKA in a time-dependent fashion [3, 7]. Furthermore, since several factors, including the femoral component, patellar height, and joint line, can influence deterioration of the patella cartilage, the time factor cannot be considered on its own. Second, since this study was not a comparative study with the group that had undergone patellar resurfacing, it is difficult to guarantee that non-resurfacing shows better results than resurfacing. Therefore, a comparative, randomized, prospective study is required to confirm that patellar retention can also provide favorable results regardless of the cartilage status of the patella. Third, other types of femoral components, such as cruciate retaining or single radius, were not considered. As reported, the trochlear geometry of the femoral component may vary with each design, and some are incompatible with the native patella [39, 40]. Therefore, the results may differ in patients treated with other types of femoral components. However, in this study, a single senior surgeon performed the surgery using the same implant with a patella-friendly design and gradual radius to minimize bias between patients. Finally, a female predominance was observed in this study. Thus, the same outcomes may not apply to populations with different sex ratios. However, OA is known to be more common in Asian women [41]. In particular, South Korean women have a five- to sevenfold higher rate of knee arthroplasty [42].

## Conclusion

The degree of intraoperatively identified patellar cartilage loss did not affect the short-term outcomes following primary TKA without patellar resurfacing.

## Data Availability

The datasets generated and/or analyzed during the current study are not publicly available, but they are available from the corresponding author on reasonable request.
